# Using Survey Data to Identify Opportunities to Reach Women with An Unmet Need for Family Planning: The Example of Madagascar

**DOI:** 10.3934/publichealth.2016.3.629

**Published:** 2016-08-23

**Authors:** Dominique Meekers, Raseliarison Ratovonanahary, Tokinirina Andrianantoandro, Hiangotiana Randrianarisoa

**Affiliations:** 1 Department of Global Community Health and Behavioral Sciences, Tulane School of Public Health and Tropical Medicine, New Orleans, USA; 2 Coalition Malagasy pour le Renforcement du Système de Santé (COMARESS), Antananarivo, Madagascar; 3 Independent consultant, Antananarivo, Madagascar; 4 Cabinet d'Etudes Recherche et Appui au Devéloppement Social et Economique (RADSE), Antananarivo, Madagascar

**Keywords:** family planning, unmet need, health services, service integration, sub-Saharan Africa

## Abstract

In several African countries fertility levels have stagnated or increased slightly. However, many women still report an unmet need for family planning. Therefore achieving further fertility declines requires programs that increase demand for family planning, but that also address the existing unmet need. One way to improve contraceptive access in a cost-effective manner might be to integrate family planning services into other existing health services. This paper analyzes secondary data from the 2012–2013 Millennium Development Goals (MDG) survey in Madagascar to estimate the number of women with an unmet need for family planning that might benefit from integrating family planning services into other health services. In Madagascar, one third of the demand for family planning is not met; an estimated 820,000 women have an unmet need for family planning. A substantial portion of these women can be reached by integrating family planning services into existing maternal and child health services. Health providers are uniquely positioned to help address method-related reasons for non-use of family planning, such as concerns about health problems and side-effects. Given the large unmet need for family planning, programs should not exclusively focus on increasing the demand for family planning, but also seek new ways to address the existing unmet need. Our study illustrates that simple analyses of existing health survey data can be an important tool for informing the design of programs to tackle this unmet need.

## 1. Introduction

A large body of public health research focuses on identifying risk factors for various health problems and on estimating the prevalence of health problems, the use of health services, etc. This type of research has been invaluable for the development of more effective public health programs and for identifying the key target groups for such programs. However, in many cases public health programs need not only prevalence estimates, but also estimates of population totals. For example, in the case of family planning, several countries struggle with unanticipated stockouts of contraceptive supplies. Data on the total number of users of various family planning methods in a country can help estimate the amount of contraceptive supplies that need to be produced or imported, which can help improve contraceptive security by reducing the occurrence of such stockouts. Countries that seek to improve access to contraceptives could potentially do this by scaling up certain delivery mechanisms (e.g., by increasing the number of family planning clinics). Another way to improve contraceptive access in a cost-effective manner might be to integrate family planning services into other existing health services. To make informed decisions about investments to expand the contraceptive access, it is important to know how many women would benefit from the planned changes. Although estimates of population totals of potential beneficiaries are rarely available, they can be estimated using existing health survey data.

This paper analyzes data from a recent health survey in Madagascar to investigate the potential for reaching women with an unmet need for family planning by integrating family planning services into other existing health services. Specifically, we investigate which health services these women are most likely to use, and estimate the total number of women with an unmet need for family planning that could potentially be reached through various types of health services. Using the example of Madagascar, this paper illustrates the estimation of population totals (potential program beneficiaries) using standardized health surveys, such as the Demographic and Health Surveys. In addition, we examine the correlates of method-related reasons for not using family planning, as health providers may be in a position to address method-related concerns and problems. These types of simple analyses can be used to guide the design of integrated family planning programs that can help address the unmet need for family planning.

## 2. Background

In sub-Saharan Africa fertility levels started to decline by the 1990s. Initially, fertility declined substantially, but recently fertility levels have stagnated in a number of countries (e.g. Burkina Faso, Ghana, Kenya, Nigeria, Senegal) or even increased slightly (e.g., Cameroon, Niger, Madagascar, Mozambique, Zambia, Zimbabwe). In all these countries, the Total Fertility Rate (TFR) leveled off above four children per woman. It is tempting to assume that fertility levels stagnated because people prefer large families and because the current demand for family planning has already been met. However, in most countries a substantial fraction of women report an unmet need for family planning. This implies that achieving further fertility declines requires not only programs that generate more demand for family planning, but also programs that more effectively address the existing unmet need for family planning.

Madagascar is a good example. During the last two decades, Madagascar experienced a notable decline in fertility. The Total Fertility Rate declined steadily from 6.1 children per woman in 1992 to 5.2 in 2003–4, and to 4.8 in 2008–9. Initially, fertility levels declined for all age groups but between 2003–4 and 2008–9 fertility levels declined only for women under age 35 [1]–[4]. After 2009, fertility levels stagnated and by 2012–13 the TFR had increased slightly to 5.0 children per woman [5].

The decline in fertility between 1992 and 2009 corresponds with large increases in knowledge and use of modern family planning. Knowledge of at least one modern contraceptive method among women aged 15–49 increased from 56.9% in 1992 to 93.7% in 2008–9, but decreased slightly to 88.8% by 2012–13. Ever use of modern contraceptives also rapidly increased from 8.3% in 1992 to 40.8% in 2009, but only increased modestly afterward, reaching 44.1% by 2012–13. Similarly, current use of modern contraceptives increased from 3.5% in 1992 to 23.0% in 2009, but reached only 26.9% by 2012–13 [2],[4],[5].

Because the demand for family planning has increased, the limited increase in modern contraceptive use in recent years is a concern. A substantial fraction of women still have an unmet need for family planning [6]. In 2012–13, one out of every seven married women reported having an unmet need for family planning. Specifically, 6.7% of all married women had an unmet need for spacing and 8.2% an unmet need for family limitation. Calculated as a percentage of the total demand, 33.4% of the total demand for family planning among married women has not been met [5].

Considering that one third of the total demand for family planning is not being met in Madagascar, it is important to explore other opportunities for reaching those women whose family planning needs are not being met. To increase contraceptive access in Madagascar, the United States Agency for International Development (USAID) has been considering integrating family planning services into other existing health services. To assess whether investing in such an integration of services can be justified, and to make evidence-based decisions about the types of health services that should be used, data on the potential number of beneficiaries are needed.

## 3. Data and Methods

This study aims to identify opportunities for reaching women who have an unmet need for family planning. Specifically, we identify which types of health services women with an unmet need for family planning are most likely to use and estimate the total number of women with an unmet need for family planning that could potentially be reached by integrating family planning into different types of health services.

### 3.1. Data

We use de-identified secondary data from the 2012–13 Millennium Development Goals (MDG) survey, which was implemented by the Institut National de la Statistique de Madagascar (INSTAT) in accordance with the required ethical guidelines [5],[7]. The survey used a two-stage stratified cluster sample (n = 15,675). The questionnaire collected information on use of several health services, including prenatal care, postnatal care, delivery assistance by trained providers, use of health centres, and visits by community-based family planning agents. Half of the respondents (n = 7,666) received a longer version of the questionnaire that covered additional topics, including injections and HIV testing.

### 3.2. Variables

Based on the available data, we calculated a series of dichotomous variables that indicate whether or not the respondent had:

- Visited a health centre in the past 12 months
- Been visited by a community-based agent who spoke about family planning during the past 12 months
- Received any type of injection during the past 12 months
- Received prenatal care in the public sector (for the last birth)
- Received prenatal care in the private sector (for the last birth)
- Delivered her last child in a public sector facility
- Delivered her last child in a private sector facility
- Received prenatal care from a trained health professional (last birth)
- Received delivery assistance from a trained health professional (last birth)
- Received postnatal care from a trained health professional (last birth)
- Received postnatal care from a trained health professional within 6 weeks after the last birth
- Obtained DPT vaccination for the last birth
- Been tested for HIV
- Been tested for HIV in the past 12 months
- Been tested for HIV as part of prenatal care for her last birth
- Been tested for HIV as part of postnatal care for her last birth
- Obtained treatment for a sexually transmitted infection (STI)
- Obtained treatment for an STI in the public sector
- Obtained treatment for an STI in the private sector

Overall contact with the health sector was measured using two dichotomous variables that indicate whether or not the respondent: (1) ever used any of the following health services: visited a health centre; met with a community-based agent who spoke about family planning; injections; obtained prenatal care/delivery assistance/postnatal care for the last birth by a trained health professional; obtained a DPT vaccination for the last birth; had an HIV test; or had STI treatment; (2) used any of the following services during the past 12 months: visited a health centre; met with a community-based agent who spoke about family planning; injections; had prenatal care/delivery assistance/postnatal care for the last birth by a trained health professional; obtained DPT vaccination for the last birth; or had an HIV test.

Finally, we calculated a dichotomous variable that indicates whether the respondent had an unmet need for family planning. This variable was based on the definition of unmet need used in the Demographic and Health Surveys, and differs slightly from the one used in the MDG survey report [6],[8],[9].

The survey also asked non-users who did not want a child in the next two years to indicate their reasons for not using family planning. We used this information to calculate four dichotomous variables indicating whether or not the respondent reported method-related reasons for non-use (health problems; concerns about side-effects; a lack of access or distance; and the method being too expensive).

### 3.3. Methods

The first part of our analysis aimed to identify the types of health services that could be used to potentially reach those women who have an unmet need for family planning. Specifically, we calculated the weighted percentage of women with an unmet need for family planning who report having used various types of health services. All estimates were calculated using STATA's *svy* procedures, which correct for the complex nature of the sample [10].

The second part of our analysis estimated the total number of women with an unmet need for family planning who reported having used each type of health service. This number is a good proxy for the number of new contraceptive users that could potentially be generated by the integration of family planning into those other health services.

To estimate the total number of women with an unmet need for family planning we used STATA's “*svy*: total” command, which is the simple weighted population total for complex samples that include stratification and clustering [11],[12]. The simple weighted estimator of the population total, $\hat{Y}$, also called the Horvitz-Thompson estimator, equals:

$$\hat{Y}=\sum_{h=\text{1}}^{L}\sum_{i=\text{1}}^{n_{h}}\sum_{j=\text{1}}^{m_{hi}}w_{hij}y_{hij}$$

Where *h* represents the strata in the survey sample (*h* = 1, …, *L*), *i* represents the clusters within the strata (*i* = 1, 2, …, *n_h_*), where *n_h_* is the total number of clusters in sample stratum *h*, and *j* represents the respondents in the sample, where *m_hi_* is the number of survey respondents in cluster (*h, i*). *y_hij_* is the variable for each survey respondent and *w_hij_* is the sampling weight for that respondent.

The estimate of the number of people in the total population with the specific attribute of interest—in our case, who had an unmet need for family planning—is the sum of the weights:

$$\hat{M}=\sum_{h=\text{1}}^{L}\sum_{i=\text{1}}^{n_{h}}\sum_{j=\text{1}}^{m_{hi}}w_{hij}$$

Because the sampling weights of the MDG survey have been standardized, the weights needed to be expanded back to the population scale in order to get an unbiased estimate of the population total. To achieve this, the weight for each case was multiplied by the ratio of total population of women aged 15–49 in Madagascar over the number of women aged 15–49 in our sample [12],[13]. The total number of women aged 15–49 has been estimated at about 5.2 million. It is noted that due to the design effect of the complex sample, the weighted estimates of the total number of women with an unmet need for family planning have a larger variance than the one expected for a simple random sample of the same size [12]. Consequently, the confidence intervals can be quite large, particularly for indicators pertaining to smaller subsamples of women.

The third part of our analysis examines the correlates of various method-related reasons for non-use of family planning (health problems, fear of side-effects, lack of access, cost), as these are issues that could potentially be addressed through the integration of family planning services into other existing health services. We use logistic regression analysis to test the association between the reasons for non-use and indicators of sociodemographic status (age group, marital status, and children ever born), economic status (urban residence, education, wealth quintile), ever use of modern contraceptives, and indicators of mass media exposure to family planning messages in the past year (on radio, TV, and newspapers/magazines).

## 4. Results

### 4.1. Sample Characteristics

Table 1 shows the socioeconomic and demographic characteristics of our sample. The first column shows the characteristics of the sample of women aged 15–49 (n = 15,675). Tananarive and Fianarantsoa are the most populous regions with 31% and 19% of the women in the sample. Overall, 20% of women live in urban areas and 36% have attended secondary or higher education. Almost two thirds of the women are married (65%), and over 60% have at least two children.

The second column in Table 1 shows the characteristics of the subsample of women who have an unmet need for family planning (n = 2,499). Women with an unmet need for family planning have similar characteristics, but are somewhat less likely to reside in urban areas (27%) or to have secondary or higher education (29%). They also tend to be somewhat poorer and older than women in the general population. As anticipated, the large majority of women with an unmet need for family planning are married (85%) and have two or more children (77%).

**Table 1.** Characteristics of women aged 15–49 and of women with an unmet need for family planning.

	All women aged 15–49%	Women with an unmet need for FP %
(Former) Province		
Tananarive	31.2	25.9
Fianarantsoa	19.3	19.6
Tamatave	16.1	15.6
Majunga	11.7	14.5
Tulear	14.0	13.4
Diego-Suarez	7.7	11.0
Residence		
Rural	79.6	82.7
Urban	20.4	17.3
Education ^1^		
None	21.0	27.0
Primary	42.9	43.7
Secondary+	36.1	29.3
Wealth Quintiles ^2^		
Q1 (Poorest)	16.2	19.9
Q2	17.9	21.0
Q3	19.3	19.8
Q4	21.4	18.8
Q5 (Wealthiest)	25.2	20.6
Age Group		
15–19	21.3	13.5
20–24	17.9	16.2
25–29	15.8	15.9
30–34	14.5	15.8
35–39	12.7	15.4
40–44	10.1	14.9
45–49	7.7	8.4
Marital Status ^3^		
Not married	34.7	14.7
Married	65.3	85.3
Number of children		
None	22.2	10.7
One	17.0	12.6
Two or three	30.0	30.2
Four +	30.8	46.5
**Total**	**100% (n = 15,675)**	**100% (n = 2,499)**

Note: Weighted percentages; unweighted n of cases. Source: Institut National de la Statistique de Madagascar (INSTAT), 2014 (^1^ n = 15,671; 2,497; ^2^ n = 15,671; 2,499; ^3^ n = 15,673; 2,499).

### 4.2. Use of Health Services by Women with an Unmet Need for Family Planning

**Figure 1.**
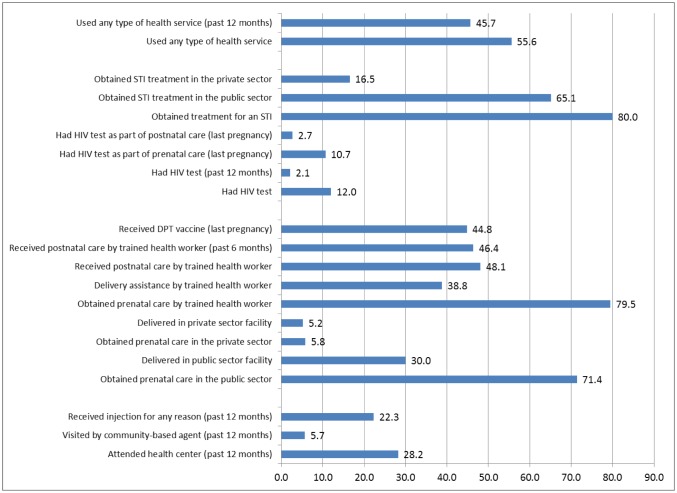
Percentage of women with an unmet need for family planning who report having used various health services. Source: Institut National de la Statistique de Madagascar (INSTAT), 2014.

To assess the potential for integrating family planning services into other health services we wanted to identify the types of health services that were used most often by women who reported having an unmet need for family planning. Figure 1 shows the percentage of women with an unmet need for family planning who reported having used different types of health services. The results indicate that 56% of women with an unmet need had visited at least one type of health service and that 46% did so during the 12 months preceding the survey.

About one out of every four women with an unmet need for family planning attended a health centre in the past year (28%) and one in five (22%) received an injection. Only 6% of women with an unmet need reported that they were visited by a community health agent who spoke about family planning during the past year.

A large percentage of women with an unmet need for family planning reported that a trained health worker provided maternal and child health care for their last birth. Specifically, 80% received prenatal care by a trained health professional, 48% received postnatal care by a trained health professional, and 39% reported that a trained health provider assisted with their last delivery. Most of these services were obtained in the public sector: 71% of women with an unmet need for family planning received prenatal care for their last birth in the public sector and 30% reported that their last delivery took place in a public sector facility.

About one out of every eight women with an unmet need for family planning had been tested for HIV (12%), often as part of prenatal care for their last child (11%). Although few women with an unmet need for family planning reported having had an STI (n = 23), most of them reported that they sought treatment (80%), usually in the public sector (65%).

### 4.3. Estimated Number of Women with an Unmet Need for Family Planning Who can be Reached through other Health Services

**Figure 2.**
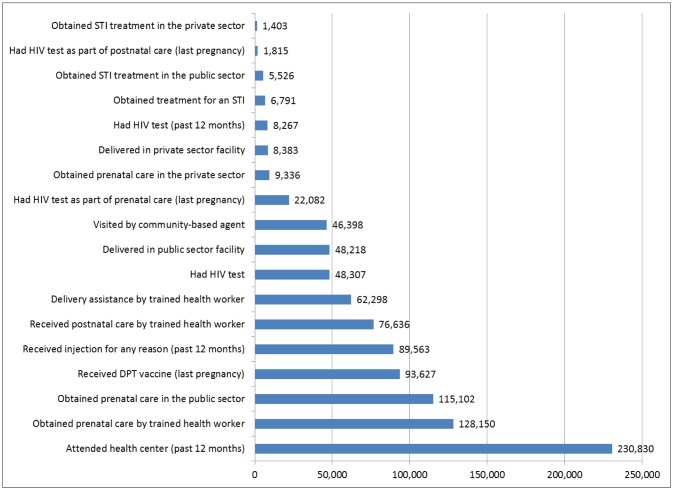
Estimated number of women with an unmet for family planning, among clients of various health services. Source: Institut National de la Statistique de Madagascar (INSTAT), 2014.

We estimated that 820,357 women in Madagascar had an unmet need for family planning (with a confidence interval of 763,641 to 877,073). Figure 2 shows the number of women with an unmet need for family planning that could potentially be reached by integrating family planning services into other health services. An estimated 230,830 women (202,447–259,214) with an unmet need for family planning visited a health centre in the past year. As anticipated, maternal and child health services appear to provide a good opportunity for reaching large numbers of women with an unmet need for family planning. Specifically, an estimated 128,150 women (108,545–147,754) with an unmet need for family planning obtained prenatal care from a trained health provider and 93,627 (77,616–109,638) reported obtaining DPT vaccinations for their child. Of the women with an unmet need for family planning, 89,563 (76,338–102,789) reported receiving an injection in the past year. The data also suggest that HIV testing services, STI treatment services, and various types of private sector health services provide relatively few opportunities for reaching women with an unmet need for family planning.

### 4.4. Reasons for Non-use of Family Planning

Descriptive results show that geographic and financial access are not important reasons for non-use of family planning. Among non-users who did not want a child in the next two years, only 1.4% reported lack of access as one of their reasons for non-use, while the cost of family planning was a reason for non-use for 4.0% of non-users. However, 13.2% reported health problems as their reason for non-use and 15.2% reported fear of side-effects as the reason.

Table 2 shows logistic regression results of the relative odds that respondents gave each method-related reason for not using family planning (among women who did not want a child in the next two years). The relative odds that women reported health problems as their reason for non-use does not vary by type of place of residence or level of education. However, women in the top two wealth quintiles are significantly more likely than poor women to report health concerns as their reason for non-use (OR = 1.83 for Q4 and OR = 1.77 for Q5). Similarly, older women are much more likely than teenage women to report health concerns (OR = 1.71 for aged 30–39; OR = 1.70 for ages 40–49). It is important to note that women who have already used modern family planning in the past are nearly twice as likely as other women to report non-use due to health concerns (OR = 1.96). Further research is needed to investigate whether this reflects bad experiences with use of modern family planning, or whether these women have reached a stage of life in which they are considering a long-term or permanent method. Concern about side-effects as a reason for non-use increases with level of education (OR = 1.61 for women with primary education; OR = 1.81 for women with secondary or higher education). However, side-effects are less of a concern for wealthy women (OR = 0.62 for Q4), women in their forties (OR = 0.64), and women who have previously tried modern methods (OR = 0.65).

The odds of non-use due to a lack of access vary little across subgroups. However, women in the top three wealth quintiles are less likely than poor women to report a lack of access as their reason for non-use. The odds of not using family planning because it is too expensive do not vary much by socioeconomic status. Only women in the wealthiest quintile are significantly less likely than poor women to report non-use due to the cost of the methods.

**Table 2.** Relative odds of having various method-related reasons for not using family planning (among women who do not want a child in the next two years).

	Health concerns	Side-effects	Lack of access	Too expensive
Residence				
Rural	1.00	1.00	–	1.00
Urban	1.03 (0.73–1.46)	1.34 (0.92–1.94)	–	0.79 (0.36–1.72)
Education				
None	1.00	1.00	1.00	1.00
Primary	1.15 (0.86–1.52)	1.61 (1.26–2.06) ***	1.26 (0.61–2.60)	1.02 (0.68–1.51)
Secondary+	0.87 (0.63–1.21)	1.81 (1.29–2.54) ***	0.54 (0.13–2.16)	1.38 (0.74–2.59)
Wealth Quintiles				
Q1 (Poorest)	1.00	1.00	1.00	1.00
Q2	1.05 (0.74–1.48)	0.88 (0.64–1.22)	0.60 (0.31–1.14)	0.92 (0.43–1.96)
Q3	1.32 (0.90–1.95)	0.80 (0.57–1.13)	0.27 (0.12–0.60) ***	0.78 (0.40–1.55)
Q4	1.83 (1.22–2.75) ***	0.62 (0.44–0.87) ***	0.38 (0.13–1.11) *	0.79 (0.37–1.68)
Q5 (Wealthiest)	1.77 (1.12–2.78) **	0.82 (0.53–1.26)	0.33 (0.10–1.07) *	0.35 (0.14–0.86) **
Age Group				
15–19	1.00	1.00	1.00	1.00
20–29	1.18 (0.74–1.89)	0.97 (0.68–1.40)	0.78 (0.39–1.59)	1.41 (0.67–2.98)
30–39	1.71 (1.05–2.79) **	1.17 (0.78–1.75)	1.16 (0.44–3.05)	2.08 (0.95–4.57) *
40–49	1.70 (1.04–2.78) **	0.64 (0.42–0.96) **	0.39 (0.11–1.36)	1.20 (0.49–2.94)
Current married				
No	1.00	1.00	1.00	1.00
Yes	3.61 (2.58–5.04) ***	3.60 (2.76–4.69) ***	1.71 (0.94–3.12)*	1.58 (1.01–2.48) **
Number of children				
None	1.00	1.00	1.00	1.00
One	0.78 (0.39–1.56)	0.95 (0.54–1.65)	1.37 (0.29–6.50)	1.68 (0.33–8.50)
Two or three	0.89 (0.45–1.77)	0.94 (0.52–1.67)	1.23 (0.23–6.61)	2.29 (0.51–10.39)
Four+	1.01 (0.53–1.92)	1.08 (0.59–1.97)	0.82 (0.15–4.44)	1.42 (0.25–8.23)
Ever used modern contraceptives				
No	1.00	1.00	1.00	1.00
Yes	1.96 (1.56–2.47) ***	0.65 (0.52–0.80) ***	0.77 (0.33–1.80)	0.98 (0.47–2.05)
Exposed to FP messages on radio in past year				
No	1.00	1.00	1.00	1.00
Yes	1.11 (0.78–1.57)	0.92 (0.68–1.24)	0.49 (0.17–1.43)	0.65 (0.37–1.14)
Exposed to FP messages on TV in past year				
No	1.00	1.00	–	1.00
Yes	1.23 (0.82–1.84)	0.73 (0.42–1.26)	–	0.29 (0.11–0.81) **
Exposed to FP messages in print in past year				
No	1.00	1.00	–	1.00
Yes	1.33 (0.66–2.72)	0.81 (0.35–1.88)	–	2.65 (0.79–8.87)
Constant	0.03 (0.01–0.05) ***	0.07 (0.04–0.13) ***	0.03 (0.01–0.10) ***	0.02 (0.00–0.07) ***
N of cases	5,169	5,169	5,182	5,169

Source: Institut National de la Statistique de Madagascar (INSTAT), 2014.

* *p* < 0.10; ** *p* < 0.05; *** *p* < 0.01; (–) variable dropped because one of the categories perfectly predicted failure.

## 5. Discussion

In Africa, notable fertility declines have occurred since the early 1990s. However, in recent years fertility levels have stabilized in several countries, including Madagascar. In Madagascar, the TFR has declined gradually since the early 1990s, but has now levelled off at roughly five children per woman. Use of modern contraceptive use also increased rapidly since the 1990s, but is now increasing only very modestly [1]–[4]. At first glance, these findings appear to suggest that fertility levels are stagnating simply because people prefer to have large families and because their demand for family planning is now being met. However, a closer look at the data shows that this is not the case: A considerable percentage of women continue to have an unmet need for family planning. Hence, it is important to identify opportunities for programs to try and address this unmet need for family planning. This study has analysed data from the 2012–13 Madagascar MDG survey to identify potential opportunities for reaching women with an unmet need. Specifically, we have estimated the number of potential beneficiaries that one might reach by integrating family planning services into different types of existing health services, and have examined method-related reasons for non-use of family planning that could potentially be addressed through such integration.

Our results have several important implications. The finding that women who have an unmet need for family planning tend to be similar to women in the general population implies that unmet need is not something that mostly affects the poor, as is often assumed. Hence, programs for integrating family planning into other health services should target not only poor women, but rather all women of reproductive age.

In Madagascar, 16% of all women aged 15–49 report having an unmet need for family planning and one third of the total demand for family planning among married women is not being met [5],[6]. We estimate that this translates to over 820,000 women. Our results indicate that that many of these women could potentially be reached through existing health services that they are already using. Almost half of these women with an unmet need attended a health centre during the past 12 months, for various reasons. Women with an unmet need are more likely to use maternal and child services than any other type of health service. Estimates based on the MDG survey data indicate that over 128,000 women with an unmet need for family planning received prenatal care from a trained provider, over 115,000 obtained prenatal care in the public sector, and nearly 94,000 obtained DPT vaccinations for their youngest child.

These results imply that the integration of family planning services into existing maternal and child health services has considerable potential for reaching a large group of women with an unmet need for family planning. In contrast, integrating family planning services into HIV testing services or STI treatment services has little potential because only a small group of women use such services.

Although lack of access to family planning methods and the cost of the methods are infrequently mentioned as the reasons for non-use, trained health providers are uniquely positioned to address much more important obstacles to use of family planning, such as concerns about health problems and side effects. This implies that for the integration of family planning into existing health services to be effective, health providers should be trained to address women's questions and concerns about a range of family planning methods, and to offer them the method that best meets their needs. In the case of Madagascar, particular attention will need to be paid to the concerns of older women, but also to educated and well-to-do women. It is possible that these latter women are more concerned about their health in general, which may lead them to also be more concerned about the potential health and side-effects of family planning. The finding that non-use due to health problems is more common among women who previously used modern family planning indicates that it will be important for providers to enquire about women's experiences with family planning methods and to either address any lingering concerns they may have or to offer them different contraceptive options.

Given that many of the African countries that are experiencing stagnating fertility levels still have a considerable unmet need for family planning, it is important to recognize that programs should not exclusively focus on increasing the demand for family planning, but also seek new ways to address the existing unmet need for family planning. Our study has illustrated how simple analyses of existing health survey data can be used to estimate the number of potential beneficiaries that might result from integrating family planning services into different types of existing health services. Similar analyses can help other countries decide whether the integration of services is a viable option to help address the unmet need for family planning.
